# Effects of arthroscopic vs. mini-open rotator cuff repair on function, pain & range of motion. A systematic review and meta-analysis

**DOI:** 10.1371/journal.pone.0222953

**Published:** 2019-10-31

**Authors:** Goris Nazari, Joy C. MacDermid, Dianne Bryant, Neha Dewan, George S. Athwal

**Affiliations:** 1 School of Physical Therapy, Faculty of Health Science, Western University, London, ON, Canada; 2 Collaborative Program in Musculoskeletal Health Research, Bone and Joint Institute, Western University, London, ON Canada; 3 Roth McFarlane Hand and Upper Limb Centre, St. Joseph’s Hospital, London, ON, Canada; 4 Department of Health Sciences, Lakehead University, Canada; University of Oxford, UNITED KINGDOM

## Abstract

**Objective:**

To assess the effectiveness of arthroscopic versus mini-open rotator cuff repair on function, pain and range of motion at 3-, 6- and 12-month follow ups.

**Design:**

Systematic review and meta-analysis of randomized controlled trials.

**Setting:**

Clinical setting.

**Participants:**

Patients 18 years and older with a rotator cuff tear.

**Intervention/Comparison:**

Arthroscopic/mini-open rotator cuff repair surgery followed by post operative rehabilitation.

**Main outcome measures:**

Function and pain.

**Results:**

Six RCTs (n = 670) were included. The pooled results, demonstrated no significant difference between arthroscopic and mini open approach to rotator cuff repair on function (very low quality, 4 RCTs, 495 patients, SMD 0.00, 3-month; very low quality, 4 RCTs, 495 patients, SMD -0.01, 6-month; very low quality, 3 RCTs, 462 patients, SMD -0.09, 12-months). For pain, the pooled results, were not statistically different between groups (very low quality, 3 RCTs, 254 patients, MD -0.21, 3-month; very low quality, 3 RCTs, 254 patients, MD -0.03, 6-month; very low quality, 2 RCTs, 194 patients, MD -0.35, 12-months).

**Conclusion:**

The effects of arthroscopic compared to mini-open rotator cuff repair, on function, pain and range of motion are too small to be clinically important at 3-, 6- and 12-month follow ups.

## Introduction

Across the general population, rotator cuff tears impact 1 in 5 individuals, and 1 in 3 of those with shoulder symptoms[1]. Rotator cuff tears are more prevalent in older adults, those involved in heavy labor, males as well as individuals with previous history of injury[1]. Studies have demonstrated that surgical interventions including mini-open or arthroscopic repairs to offer satisfactory outcomes [2–4]. The mini-open has been considered the gold standard technique, costs significantly less, and proved to attain good to excellent outcomes in 90% of patients [5–8]. On the other hand, factors such as lower postoperative pain, quicker recovery time, and superior cosmetic results have steered surgeons’ preferences to choosing an arthroscopic technique based on the to emerging evidence [9–11]. However, there is no consensus on whether one technique offers superior outcomes.

To date, three systematic reviews (SRs) have examined the effectiveness of clinical outcomes in patients with rotator cuff tears undergoing arthroscopic vs mini-open rotator cuff repairs [2–4] The Shan et al. (2014) review of 12 studies (3 RCTs, 9 observational), and the Huang et al. (2016) review of 18 studies (4 RCTs, 14 observational), both concluded that there were no differences in outcomes between the arthroscopic and mini-open rotator cuff repair techniques [2–3]. However, in these reviews, studies were pooled irrespective of their design (RCT and observational), which greatly limits our confidence in its effect estimates [2–3] Furthermore, the risk of bias in the included studies were not assessed. A third review by Ji et al. (2015), included 5 RCTs and again concluded that there were no differences in outcomes at the end of follow-up between the arthroscopic and mini-open rotator cuff repair techniques [4]

While the review by Ji et al. (2015) provides valuable insights, it has important limitations. For example, trials were pooled and meta-analyses conducted based on the last follow-up time point reported (range: 6–34 months), which might have in turn contributed to the high levels of heterogeneity in the pooled analyses [12]. The effectiveness of arthroscopic versus mini-open rotator cuff repair on outcomes function, pain and range of motion, at 3-, 6- and 12-month follow ups were not assessed. Furthermore, the review failed to provided ratings of the quality of the evidence across each outcome, according to Grading of Recommendations, Assessment, Development and Evaluation (GRADE) guidelines [13]. Therefore, the purpose of this review was to conduct a systematic review with meta-analysis that addresses the reported limitations of the aforementioned reviews.

### The objectives of this review were

1. to quantify the effects of arthroscopic versus mini-open rotator cuff repair on function, pain and range of motion at 3-, 6- and 12-month follow ups,
2. to rate the quality of the body of literature that compares the effectiveness of arthroscopic versus mini-open rotator cuff repair according to GRADE guidelines across each outcome.

## Methods

We followed the Preferred Reporting Items for Systematic Reviews and Meta-Analyses (PRISMA) and Cochrane collaboration guidelines [14–15]. (S1 PRISMA Checklist) PROSPERO registration number: CRD 42018097325.

### Eligibility criteria

Studies were included in this systematic review if the below criteria were met [2–4]:

- *Design*: randomized controlled trial (RCT) in English published in a peer reviewed journal between January 1998 –July 2019,
- *Participants*: patients 18 years and older with a rotator cuff tear,
- *Intervention/ Comparison*: trials that compared patients who underwent arthroscopic or mini-open rotator cuff repair followed by post operative rehabilitation,
- *Outcomes*: function/disability, pain and shoulder range of motion.

Studies that included patients with degenerative arthritis, rheumatoid arthritis of glenohumeral joint, adhesive capsulitis/ shoulder fractures / previous surgery, that were conference abstract and posters were excluded from this systematic review [2–4].

### Information sources

We conducted systematic electronic searches to identify relevant randomized controlled trials in MEDLINE, EMBASE, CINAHL and Google scholar from January 1998 to July 2019. Several different combinations of keywords were used, such as: “rotator cuff repair”, “randomized controlled trials”, “arthroscopic surgery”, “mini-open surgery”, “rehabilitation after arthroscopic”, “rehabilitation after mini-open”, “effectiveness of arthroscopic”, “effectiveness of mini-open”. In addition, we also performed a search in the clinical trial registers catalogues (ClinicalTrials.gov, EU registry and ISRCTN registry), and carried out a manual search of the reference lists of the previous systematic reviews and the references of all the included articles.

### Study selection

Two independent reviewers (GN and ND) carried out the systematic electronic searches in each database. Duplicate studies were identified and removed. Next, we independently screened the titles and abstracts and retrieved in full text any article marked include or uncertain by either reviewer. Finally, we conducted an independent full text review to determine final eligibility. In case of disagreement, a third reviewer; the most experienced member (JM), provided a consensus through discussion.

### Data collection process

Two independent researchers (GN and ND) extracted the data from the eligible trials. In case of disagreement, a third reviewer (JM), provided a consensus through discussion. Data extraction included the author, year, study population, sample size, age, intervention/comparison group, follow up periods, primary and secondary outcomes and the protocol for postoperative therapy. When insufficient data were presented, GN contacted the authors by email and requested further data.

### Assessment of risk of bias in individual studies

Two independent review authors (GN and ND) assessed the trials for risk of bias. In case of disagreement, a third reviewer (JM), provided a consensus through discussion. The risk of bias assessment was performed using the Cochrane Risk of Bias tool [14]. The Cochrane Risk of Bias tool is based on 7 items, random sequence generation, allocation concealment, blinding of participants and personnel, blinding of outcome assessment, incomplete outcome data, selective reporting and other bias [14]. The other bias category was defined as trials that did not include statements on sources of funding and potential sources of conflicts of interest. We then rated the adequacy of each of the seven risk of bias domains as “low”, “unclear” or “high” risk according to criteria provided in the Cochrane Handbook for Systematic Reviews of Interventions [14]. (S1 Table).

### Assessing the quality of evidence

We used the GRADE approach for systematic reviews, to assess the quality of evidence related to each outcome to summarize the extent of our confidence in the estimates of the effect[16–21]. The GRADE approach considers the risk of bias, publication bias, consistency of findings, precision, and the applicability of the overall body of literature to provide a rating of quality of evidence (high, moderate, low, or very low) per outcome [16–21].

### Summary measures

To quantify and interpret our data, a minimally clinically important difference (MCID) of 1.4 points (0–10) for pain [22], a standard deviation of 0.5 points for function [23], 11.7 degrees for active shoulder forward flexion range of motion and 4.9 degrees for active shoulder external rotation range of motion were used [24] Timing of outcome assessment were categorised as 3 months, 6-months and 12-months only.

### Subgroup analysis and exploring heterogeneity

In the presence of heterogeneity, we planned to perform the following subgroup analyses (a priori): trials at low risk of bias (low risk of bias in allocation concealment and blinding of outcome assessor if objective outcomes were used) would show a smaller effect size, size of the tendon tear and postoperative therapy received. An I^2^ estimate of at least 50% and a statistically significant Chi^2^ statistic (P = 0.10) was interpreted as evidence of a substantial problem with heterogeneity [25].

### Synthesis of results

We performed 12 meta-analyses of trials comparing arthroscopic vs mini open repair, using the outcome function, whether reported by WORC, DASH or Constant; pain, reported by VAS; and range of motion, at 3-, 6- and 12-month follow ups. We used the Review Manager 5.3 (RevMan 5.3) software to conduct our review and a random-effects model to pool outcomes. For outcomes of the same construct (function) that were measured using a different metric, we used the standardized mean difference (SMD). If all eligible trials measured an outcome using the same metric (pain, flexion and external range of motion), we used a weighted mean difference (WMD).

## Results

### Study selection

**Fig 1:**
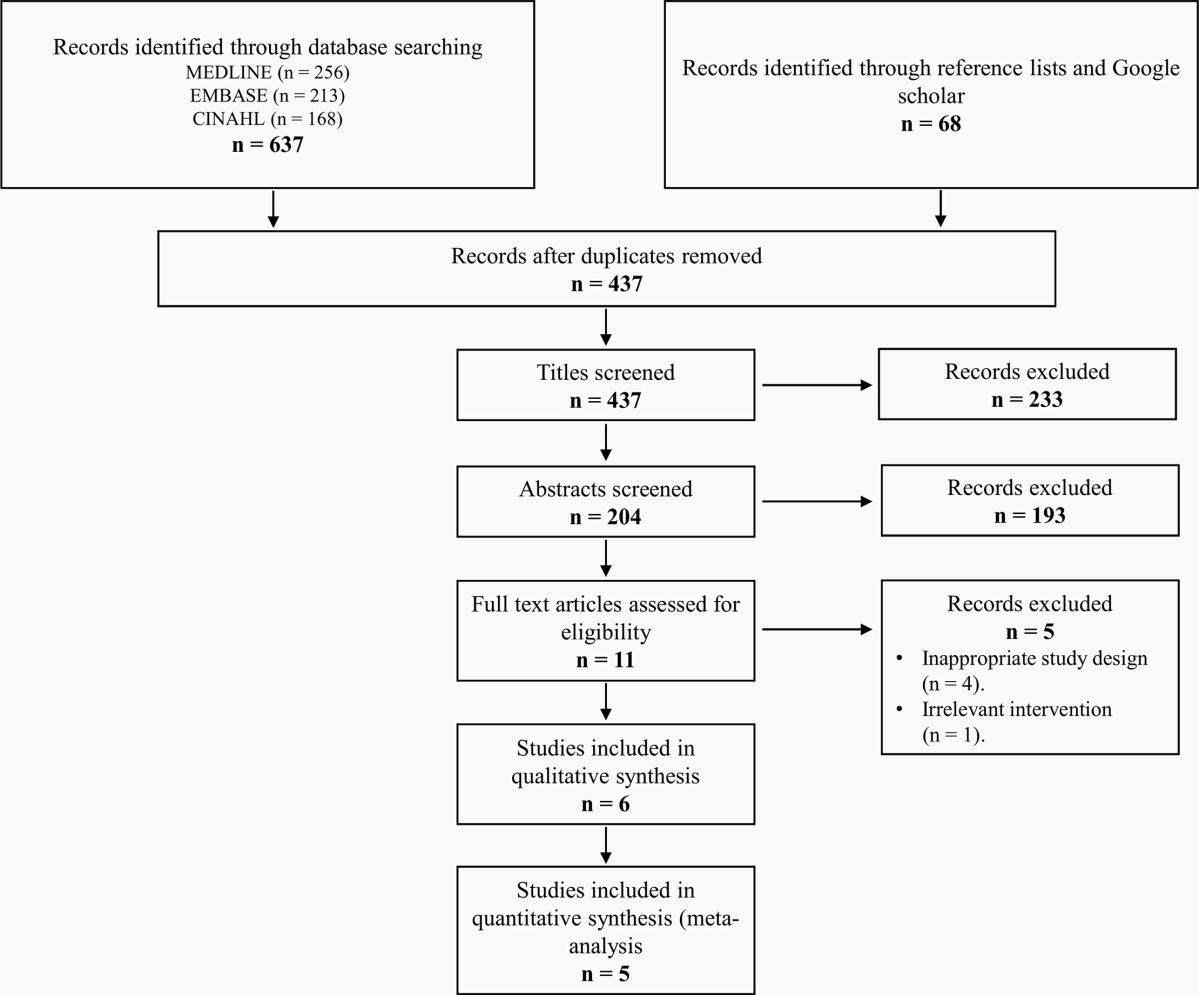
Selection of studies for inclusion in the systematic review.

Initially, our search yielded 705 publications. After removal of the duplicates, 437 articles remained and were screened using their title and abstract; leaving 11 articles selected for full text review. Of these, 6 RCTs were eligible [26–31]. The flow of studies through the selection process is presented in Fig 1.

### Study characteristics

The 6 eligible RCTs were conducted between 2011 and 2018 and included 670 patients (337 arthroscopic and 333 mini-open) [26–31]. Study size ranged from 34 to 274 patients. Trials were conducted in Japan, Germany, South Korea, Netherlands, China and Canada[26–31]. Only one out of the six trials were registered in a clinical trials register[29]. In addition, 50% of the trials (n = 3) did not include statements on sources of funding or potential sources of conflicts of interest [26,30–31]. A summary description of all the included RCTs is displayed in Table 1.

**Table 1:** Summary of included randomized controlled trials (RCTs) studies.

Study	Country	Population	Groups	Outcomes	Follow ups	Postoperative therapy (AR & MO)
Kasten et al. (2011) [27]	Germany	34 patients with isolated rupture of the supraspinatus tendon (various degrees).	AR: 17 (9 men, 8 women; 60.1 ± 8.6 yrs.). MO: 17 (12 men, 5 women; 60.1 ± 9 yrs.)	Pain levels (VAS 0–10). Function (Constant). Pain and ADL (ASES). Range of motion. Patient satisfaction.	1–12 weeks. 3, 6 months. 3, 6 months. 3, 6 months. 6 months	Four weeks abduction pillow with 30° of abduction and passive ROM exercises by a physiotherapist. Active ROM of the arm without limitations was allowed. Patients continued home exercises with a frequency of 2.5×/week in the AR group and 2.6×/week in the MO group.
Cho et al. (2012) [26]	South Korea	60 patients scheduled to undergo repair for rotator cuff tears smaller than 3 cm.	AR: 30 (17 men, 13 women; 55.5 ± 7.8 yrs.). MO: 30 (17 men, 13 women; 56.2 ± 7.9 yrs.)	Pain levels (VAS 0–10). Range of motion.	1–5 days, 2,6 weeks, 3 and 6 months. 5 days, 6 weeks, 3 and 6 months.	Wearing an abduction brace, patients engaged in pendulum and continuous passive motion machine exercises until postoperative day 5, and then passive range-of-motion exercises were started. Active range-of motion exercises were started at 6 weeks postoperatively, muscle-strengthening exercises were started at 3 months, and occupational or sports activities were started at 6 months.
Van der Zwaal et al. (2013) [30]	Netherlands	95 patients with full-thickness rotator cuff tears.	AR: 47 (29 men, 18 women; 57.2 ± 8 yrs.). MO: 48 (28 men, 20 women; 57.8 ± 7.9 yrs.)	Pain levels (VAS 0–10). Range of motion. Function (Dash, Constant).	6, 12, 26 and 52 weeks	Active exercises of the elbow, wrist, and hand were encouraged immediately. The rehabilitation protocol consisted of active abduction in the scapular plane limited to 70° and 0° of external rotation in the first 4 to 6 weeks as tolerated. After this, active range of motion exercises were started. When the patient was free of pain, scapula and rotator cuff isotonic strengthening exercises were initiated.
Zhang et al. (2014) [31]	China	108 patients with partial & full thickness rotator cuff tears.	AR: 55 (28 men, 27 women; 53.9 yrs.) MO: 53 (27 men, 26 women; 54.2 yrs.)	Pain, function, range of motion, strength, and patient satisfaction (UCLA). Pain and ADL (ASES). Muscle strength. Range of motion.	mean of 29.4 months (range 24–35 months).	Continuous passive motion machine exercise was initiated from the first day after surgery. Patients used the machine for 2 h a day until discharge from the hospital. The arc of motion of the continuous passive motion was maintained within the comfortable range, which was < 80°elevation. The gentle pendulum exercise was started from the third to fifth day and continued to the first post-operative visit, which was 3 weeks after surgery. Thereafter, the passive and active assisted range of motion exercises were started using a rope and pulley. The rehabilitation was continued for 6 months.
Liu et al. (2017) [28]	China	99 patients with full thickness rotator cuff tears.	AR: 50 (25 men, 25 women; 53.5 ± 4.3 yrs.). MO: 49 (24 men, 25 women; 52.5 ± 5 yrs.)	Pain levels (VAS 0–10). Range of motion. Function (Dash, Constant).	3 days, 1,2 weeks, 1,3,6 months and 1 year.	Wearing an abduction brace, patients engaged in pendulum and continuous passive motion machine exercises until postoperative day 5, and then passive range-of-motion exercises were started. Active range-of-motion exercises were started at 6 weeks postoperatively, muscle-strengthening exercises were started at 3 months, and occupational or sports activities were started at 6 months.
MacDermid et al. (2019) [29]	Canada	274 patients with small or medium rotator cuff tears.	AR: 138 (85 men, 53 women; 55.8 ± 8.5 yrs.). MO: 136 (80 men, 56 women; 54.6 ± 10.1 yrs.)	Function / quality of life (WORC). Pain and ADL (ASES, SPADI). Health related quality of life (SF-12). Range of motion. Strength.	6 weeks, 3,6,12,18 and 24 months.	Standardized rehabilitation protocol of progressive mobilization and strengthening, which was semi-specific and adapted to patient presentation by their physical therapist. Adherence was monitored to rehab milestones at 2 weeks, 6 weeks and 3 months postoperative by asking the physical therapist to report the date when the patient was no longer wearing their sling, when active-assisted, strengthening, and functional endurance exercises had begun. The therapist was also asking to indicate whether the patient was compliant with activity precautions throughout recovery, whether the patient was progressing as expected and to describe any off-protocol or worrisome findings.

### Risk of bias assessment in the individual studies

**Fig 2:**
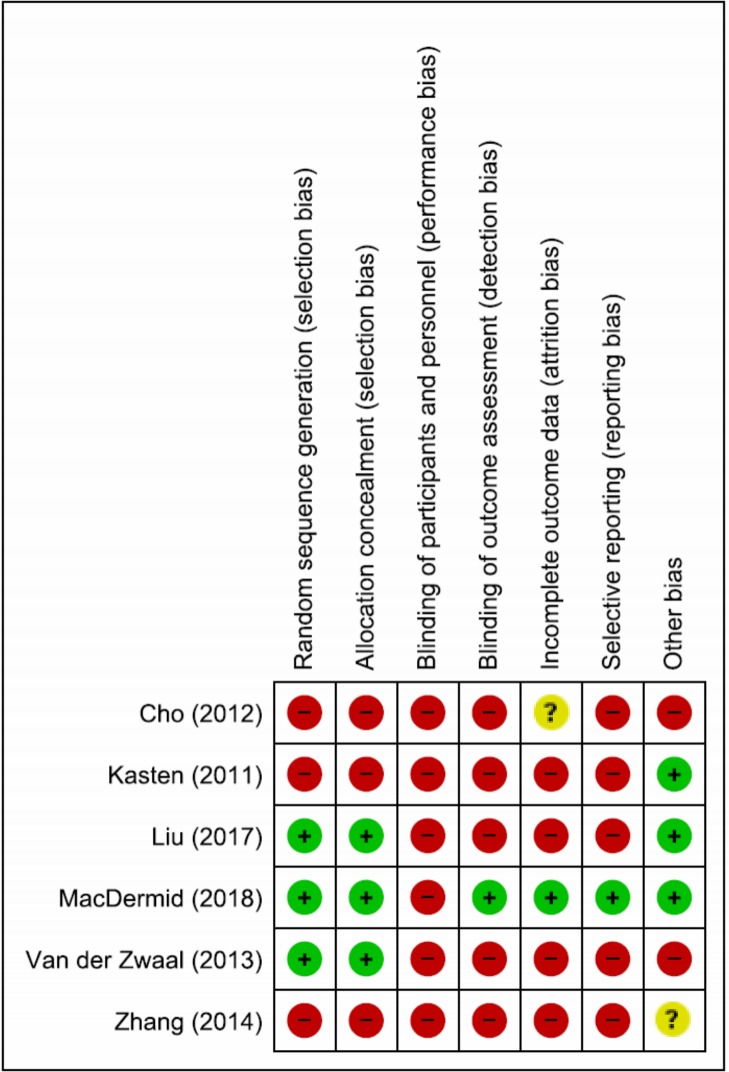
Risk of bias summary: Review authors’ judgements about each risk of bias item for each included study.

The risk of bias assessment is presented in Fig 2. Performance bias (lack of or inadequate blinding of participants who could influence how interventions, including co-interventions are performed/administered) was rated at high risk in all the included trials (n = 6)[26–31] Detection bias (lack of or inadequate blinding of participants who could influence the measurement or interpretation of outcomes) and Selective Reporting bias were rated at high risk in five trials[26, 27, 28, 30–31]. Selection bias and attrition bias (significant or imbalanced missing outcome data) were rated at high risk in three [26,27,31], and four trials respectively [26, 28, 30–31]. Other biases (RCTs with no statements on sources of funding/conflicts of interest) were rated at high risk in two trials[26,30]. Overall, all six included RCTs were rated at high risk of bias[26–31].

### GRADE Evidence Profile (EP) and Summary of Findings (SoF)

The EP (Table 2) displays a detailed quality assessment and includes a judgment of each factor that determined the quality of evidence for each outcome. The SoF tables (Tables 3–5) include an assessment of the quality of evidence for each outcome.

**Table 2:** Grade evidence profile: Arthroscopic vs mini-open for patients with rotator cuff tears.

Quality Assessment						Summary of Findings			
Outcome (No. of studies; design)	Limitations	Inconsistency	Indirectness	Imprecision	Publication Bias	Mini-open	Arthroscopic	SMD / MD (95% CI)	Quality
Function at 3 months (4 RCTs)	Serious limitations	No serious inconsistency	No serious indirectness	Serious imprecisions	Likely	247/495	248/495	SMD 0.00 (-0.18–0.18)	⊕⊝⊝⊝ very low
Function at 6 months (4 RCTs)	Serious limitations	No serious inconsistency	No serious indirectness	Serious imprecisions	Likely	247/495	248/495	SMD -0.01 (-0.23–0.21)	⊕⊝⊝⊝ very low
Function at 12 months (3 RCTs)	Serious limitations	No serious inconsistency	No serious indirectness	Serious imprecisions	Likely	231/462	231/462	SMD -0.09 (-0.28–0.09)	⊕⊝⊝⊝ very low
Pain at 3 months (3 RCTs)	Serious limitations	No serious inconsistency	No serious indirectness	Serious imprecisions	Likely	127/254	127/254	MD -0.21 (-0.91–0.50)	⊕⊝⊝⊝ very low
Pain at 6 months (3 RCTs)	Serious limitations	No serious inconsistency	No serious indirectness	Serious imprecisions	Likely	127/254	127/254	MD -0.03 (-0.25–0.19)	⊕⊝⊝⊝ very low
Pain at 12 months (2 RCTs)	Serious limitations	No serious inconsistency	No serious indirectness	Serious imprecisions	Likely	97/194	97/194	MD -0.35 (-1.02–0.31)	⊕⊝⊝⊝ very low
ROM–Forward flexion at 3 months (5 RCTs)	Serious limitations	No serious inconsistency	Serious indirectness	Serious imprecisions	Likely	277/555	278/555	MD 4.26 (-0.56–9.09)	⊕⊝⊝⊝ very low
ROM–Forward flexion at 6 months (5 RCTs)	Serious limitations	No serious inconsistency	Serious indirectness	Serious imprecisions	Likely	277/555	278/555	MD 1.39 (-2.12–4.90)	⊕⊝⊝⊝ very low
ROM–Forward flexion at 12 months (3 RCTs)	Serious limitations	Serious inconsistency	Serious indirectness	Serious imprecisions	Likely	231/461	230/461	MD 2.94 (-4.55–10.44)	⊕⊝⊝⊝ very low
ROM–External Rotation at 3 months (4 RCTs)	Serious limitations	No serious inconsistency	Serious indirectness	Serious imprecisions	Likely	261/522	261/522	MD 1.13 (-2.08–4.33)	⊕⊝⊝⊝ very low
ROM–External Rotation at 6 months (5 RCTs)	Serious limitations	No serious inconsistency	Serious indirectness	Serious imprecisions	Likely	261/522	261/522	MD 0.12 (-2.82–3.06)	⊕⊝⊝⊝ very low
ROM–External Rotation at 12 months (3 RCTs)	Serious limitations	No serious inconsistency	Serious indirectness	Serious imprecisions	Likely	231/462	231/462	MD 3.71 (0.14–7.28)	⊕⊝⊝⊝ very low

**Table 3:** Summary of findings. Arthroscopic vs open-mini repair for rotator cuff tears (3-month).

Population: patients with rotator cuff tears. Settings: inpatient clinics. Intervention: arthroscopic rotator cuff repair. Comparison: mini-open rotator cuff repair. Follow up: 3-months.			
Outcomes	SMD / MD (95% C.I.)	No of participants (studies)	Quality of the evidence (GRADE)
**Function:** DASH, Constant, WORC: (0 to 100). Higher values indicate better function	SMD 0.00 (-0.18–0.18)	495 (4 studies)	⊕⊝⊝⊝ very low^1^^,^^2^^,^^4^
**Pain:** VAS (0–10) Lower values indicate improved pain.	MD -0.21 (-0.91–0.50)	254 (3 studies)	⊕⊝⊝⊝ very low^1^^,^^2^^,^^4^
**Range of motion:** (forward flexion—degrees) Higher values indicate better range of motion.	MD 4.26 (-0.56–9.09)	555 (5 studies)	⊕⊝⊝⊝ very low^1^^,^^2^^,^^3^^,^^4^
**Range of motion:** (external rotation—degrees) Higher values indicate better range of motion.	MD 1.13 (-2.08–4.33)	522 (4 studies)	⊕⊝⊝⊝ very low^1^^,^^2^^,^^3^^,^^4^

^1^We downgraded by one level due to high risk of bias.

^2^We downgraded by one level due to a relatively small sample size.

^3^We downgraded by one level due to indirectness (surrogate outcomes).

^4^We downgraded by one level due to publication bias.

Abbreviations: VAS; visual analogue scale, DASH; Disabilities of Arm, Shoulder and Hand, WORC; western Ontario rotator cuff index, SMD; standardized mean difference, MD; mean difference, CI; confidence interval.

**Table 4:** Summary of findings. Arthroscopic vs open-mini repair for rotator cuff tears (6-month).

Population: patients with rotator cuff tears. Settings: inpatient clinics. Intervention: arthroscopic rotator cuff repair. Comparison: mini-open rotator cuff repair. Follow up: 6-months.			
Outcomes	SMD / MD (95% C.I.)	No of participants (studies)	Quality of the evidence (GRADE)
**Function:** DASH, Constant, WORC: (0 to 100). Higher values indicate better function	SMD—0.01 (-0.23–0.21)	495 (4 studies)	⊕⊝⊝⊝ very low^1^^,^^2^^,^^4^
**Pain:** VAS (0–10) Lower values indicate improved pain.	MD -0.03 (-0.25–0.19)	254 (3 studies)	⊕⊝⊝⊝ very low^1^^,^^2^^,^^4^
**Range of motion:** (forward flexion—degrees) Higher values indicate better range of motion.	MD 1.39 (-2.12–4.90)	555 (5 studies)	⊕⊝⊝⊝ very low^1^^,^^2^^,^^3^^,^^4^
**Range of motion:** (external rotation—degrees) Higher values indicate better range of motion.	MD 0.12 (-2.82–3.06)	522 (4 studies)	⊕⊝⊝⊝ very low^1^^,^^2^^,^^3^^,^^4^

^1^We downgraded by one level due to high risk of bias.

^2^We downgraded by one level due to a relatively small sample size.

^3^We downgraded by one level due to indirectness (surrogate outcomes).

^4^We downgraded by one level due to publication bias.

Abbreviations: VAS; visual analogue scale, DASH; Disabilities of Arm, Shoulder and Hand, WORC; western Ontario rotator cuff index, SMD; standardized mean difference, MD; mean difference, CI; confidence interval.

**Table 5:** Summary of findings. Arthroscopic vs open-mini repair for rotator cuff tears (12-month).

Population: patients with rotator cuff tears. Settings: inpatient clinics. Intervention: arthroscopic rotator cuff repair. Comparison: mini-open rotator cuff repair. Follow up: 12-months.			
Outcomes	SMD / MD (95% C.I.)	No of participants (studies)	Quality of the evidence (GRADE)
**Function:** DASH, WORC: (0 to 100). Higher values indicate better function	SMD -0.09 (-0.28–0.09)	462 (3 studies)	⊕⊝⊝⊝ very low^1^^,^^2^^,^^4^
**Pain:** VAS (0–10) Lower values indicate improved pain.	MD -0.35 (-1.02–0.31)	194 (2 studies)	⊕⊝⊝⊝ very low^1^^,^^2^^,^^4^
**Range of motion:** (forward flexion—degrees) Higher values indicate better range of motion.	MD 2.94 (-4.55–10.44)	461 (3 studies)	⊕⊝⊝⊝ very low^1^^,^^2^^,^^3^^,^^4^^,^^5^
**Range of motion:** (external rotation—degrees) Higher values indicate better range of motion.	MD 3.71 (0.14–7.28)	462 (3 studies)	⊕⊝⊝⊝ very low^1^^,^^2^^,^^3^^,^^4^

^1^We downgraded by one level due to high risk of bias.

^2^We downgraded by one level due to a relatively small sample size.

^3^We downgraded by one level due to indirectness (surrogate outcomes).

^4^We downgraded by one level due to publication bias.

^5^We downgraded by one level due to inconsistency.

Abbreviations: VAS; visual analogue scale, DASH; Disabilities of Arm, Shoulder and Hand, WORC; western Ontario rotator cuff index, SMD; standardized mean difference, MD; mean difference, CI; confidence interval.

### Participants

Among the eligible RCTs, one recruited patients with an isolated rupture of the supraspinatus tendon (various degrees)[27], one included patients with rotator cuff tears smaller than 3 cm[26], two included patients with full-thickness rotator cuff tears[28,30], one recruited patients with partial and full thickness rotator cuff tears[31], and one included patients with small or medium rotator cuff tears[29].

### Outcomes

Pain levels were measured using a Visual Analogue Scale (VAS)[26,27,28,30]. Function was measured using DASH[28,30], Constant[27,28,30] and WORC[29]. Range of motion, in degrees was assessed in all six trials[26–31]. The follow-up period was up to 41 months postoperatively.

### Effects on function (patient reported function)

**Fig 3:**
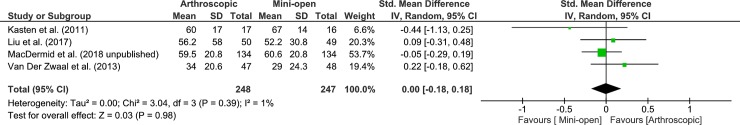
Forest plot of comparison: Arthroscopic vs Open-mini, 3 months after surgery–rotator cuff repair, outcome: Function (DASH, Constant, WORC), 4 RCTs. Higher values indicate better/improved function.

**Fig 4:**
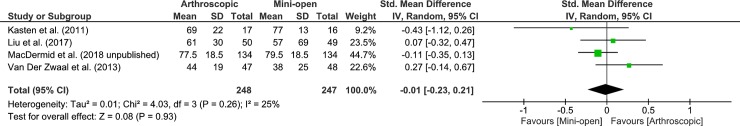
Forest plot of comparison: Arthroscopic vs Open-mini, 6 months after surgery–rotator cuff repair, outcome: Function (DASH, Constant, WORC), 4 RCTs. Higher values indicate better/improved function.

**Fig 5:**
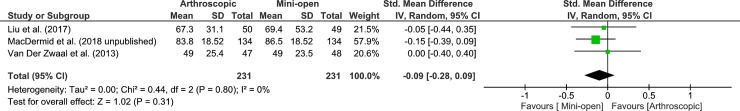
Forest plot of comparison: Arthroscopic vs Open-mini, 12 months after surgery–rotator cuff repair, outcome: Function (DASH, WORC), 3 RCTs. Higher values indicate better/improved function.

Four studies were pooled to examine the effects of arthroscopic vs mini-open on function at 3-month follow up. The pooled results, demonstrated no significant difference between arthroscopic and mini open approach to rotator cuff repair (very low quality, 4 RCTs, 495 patients, SMD 0.00, 95% CI: -0.18 to 0.18, p = 0.98, Fig 3). We found similar results at 6-month follow up, (very low quality, 4 RCTs, 495 patients, SMD -0.01, 95% CI: -0.23 to 0.21, p = 0.93, Fig 4) and at 12-month follow up, (very low quality, 3 RCTs, 462 patients, SMD -0.09, 95% CI: -0.28 to 0.09, p = 0.31, Fig 5). Heterogeneity was low at 3 and 6 months and absent at 12 months. Given that an MCID is approximately 0.5 SD[23], and that the 95% CIs at each follow up exclude the MCID of 0.5 SD, for majority of patients either approach to rotator cuff repair will result in superior functional outcomes (12 month arthroscopic mean function 66.7/100; mini-open mean function 68.3/100).

### Effects on pain (patient reported pain)

**Fig 6:**
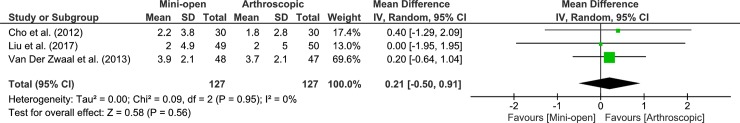
Forest plot of comparison: Arthroscopic vs Open-mini, 3 months after surgery–rotator cuff repair, outcome: Pain (VAS 0–10), 3 RCTs. Lower values indicate better/improved pain.

**Fig 7:**
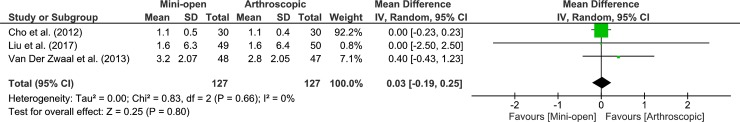
Forest plot of comparison: Arthroscopic vs Open-mini, 6 months after surgery–rotator cuff repair, outcome: Pain (VAS 0–10), 3 RCTs. Lower values indicate better/improved pain.

**Fig 8:**
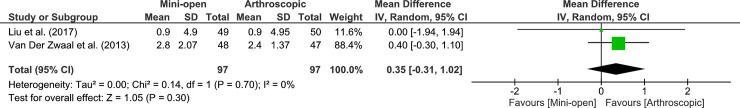
Forest plot of comparison: Arthroscopic vs Open-mini, 12 months after surgery–rotator cuff repair, outcome: Pain (VAS 0–10), 2 RCTs. Lower values indicate better/improved pain.

Three studies were pooled to examine the effects of arthroscopic vs mini-open on pain levels at 3-month follow up. The pooled results, were not statistically different between groups (very low quality, 3 RCTs, 254 patients, MD -0.21, 95% CI: -0.91 to 0.50, p = 0.56, Fig 6). We found similar results at both 6- and 12-month follow ups, (very low quality, 3 RCTs, 254 patients, MD -0.03, 95% CI: -0.25 to 0.19, p = 0.80, Fig 7; very low quality, 2 RCTs, 194 patients, MD -0.35, 95% CI: -1.02 to 0.31, p = 0.30, Fig 8) respectively. Heterogeneity was absent for all analyses. Because the 95% CIs at each follow up exclude the MCID of 1.4 points on a 10-point scale[22], it is extremely unlikely that either approach to rotator cuff repair will result in lower pain levels.

### Effects on forward flexion range of motion (performance-based function)

**Fig 9:**
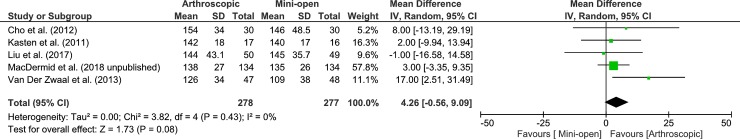
Forest plot of comparison: Arthroscopic vs Open-mini, 3 months after surgery–rotator cuff repair, outcome: ROM (Forward Flexion°), 5 RCTs. Higher values indicate better/improved ROM.

**Fig 10:**
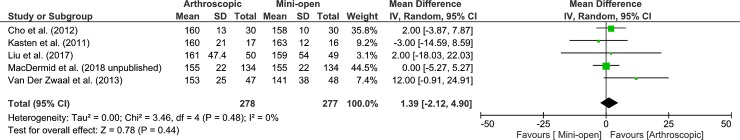
Forest plot of comparison: Arthroscopic vs Open-mini, 6 months after surgery–rotator cuff repair, outcome: ROM (Forward Flexion°), 5 RCTs. Higher values indicate better/improved ROM.

**Fig 11:**
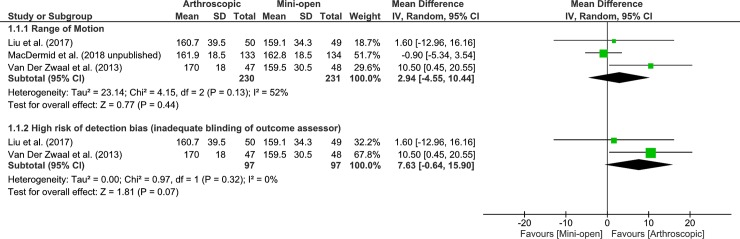
Forest plot of comparison: Arthroscopic vs Open-mini, 12 months after surgery–rotator cuff repair, outcome: ROM (Forward Flexion°), 3 RCTs. 1.1.2 Subgroup analysis by high risk of detection bias, 2 RCTs. Higher values indicate better/improved ROM.

Five studies were pooled to examine the effects of arthroscopic vs mini-open on shoulder forward flexion range of motion at 3-month follow up. The pooled results, showed no statistically significant difference between groups (very low quality, 5 RCTs, 555 patients, MD 4.26, 95% CI: -0.56 to 9.09, p = 0.08, Fig 9). Our findings were similar at both the 6- and 12-month follow ups, (very low quality, 5 RCTs, 555 patients, MD 1.39, 95% CI: -2.12 to 4.90, p = 0.44, Fig 10; very low quality, 3 RCTs, 461 patients, MD 2.94, 95% CI: -4.55 to 10.44, p = 0.44, Fig 11) respectively. Heterogeneity was absent in the analysis of 3 and 6 month follow up, and because the 95% CIs exclude the MCID of 11.7°[24], it is extremely unlikely that either approach to rotator cuff repair will result in better flexion range of motion. Heterogeneity was substantial in the analysis of 12 month follow up and our subgroup analysis of the two studies at high risk of detection bias indicated that the likely cause of substantial heterogeneity was due to inadequate blinding of outcome assessors. The one remaining MacDermid et al. (2018) study with adequate blinding of outcome assessors showed no statistically significant difference between groups (1 RCT, 267 patients, MD -0.90, 95% CI: -5.34 to 3.54, p = 0.69) and because the 95% CIs exclude the MCID of 11.7 degrees[24], it is unlikely that either approach to rotator cuff repair will result in better flexion range of motion.

### Effects on external rotation range of motion (performance-based function)

**Fig 12:**
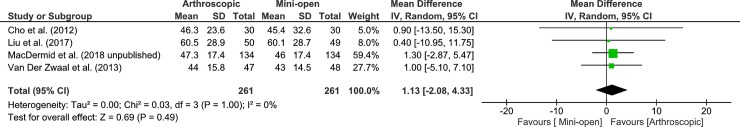
Forest plot of comparison: Arthroscopic vs Open-mini, 3 months after surgery–rotator cuff repair, outcome: ROM (External Rotation°), 5 RCTs. Higher values indicate better/improved ROM.

**Fig 13:**
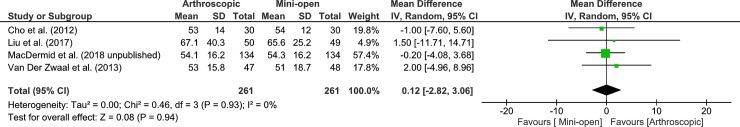
Forest plot of comparison: Arthroscopic vs Open-mini, 6 months after surgery–rotator cuff repair, outcome: ROM (External Rotation°), 4 RCTs. Higher values indicate better/improved ROM.

**Fig 14:**
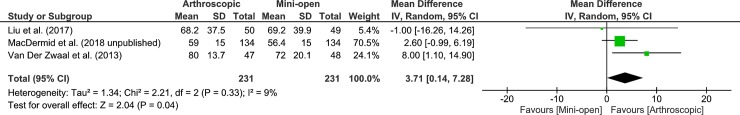
Forest plot of comparison: Arthroscopic vs Open-mini, 12 months after surgery–rotator cuff repair, outcome: ROM (External Rotation°), 3 RCTs. Higher values indicate better/improved ROM.

Five studies were pooled to examine the effects of arthroscopic vs mini-open on shoulder external rotation range of motion at 3-month follow up. The pooled results, showed no statistically significant difference between groups (very low quality, 4 RCTs, 522 patients, MD 1.13, 95% CI: -2.08 to 4.33, p = 0.49, Fig 12). Our findings were similar at the 6-month follow up (very low quality, 4 RCTs, 522 patients, MD 0.12, 95% CI: -2.82 to 3.06, p = 0.94, Fig 13). However, at 12-month follow up the pooled results showed statistically significant difference between groups (very low quality, 3 RCTs, 462 patients, MD 3.71, 95% CI: 0.14 to 7.28, p = 0.04, Fig 14). Heterogeneity was absent in the analysis of 3 and 6 month follow up and low at 12 months. Given the MCID of 4.9 degrees[24], we can confidently rule out the possibility that surgical approach will cause a difference in external rotation range of motion at 6 months. However, we are unable to make this same declaration for the results at 3 and 12 months as it remains possible that an arthroscopic approach could offer superior outcomes in terms of external rotation range of motion. More data is required to make a definitive conclusion.

## Discussions

We aimed to summarise the current evidence of the effects of arthroscopic vs mini-open rotator cuff repair on clinical outcomes. Our forest plots for flexion and external rotation range of motion outcomes displayed that when considering the results of individual studies (not pooled analyses), there is a trend, indicating that arthroscopic treatment may yield better outcomes. However, upon meta-analysis, we found no clinically important differences in function, pain, flexion or external rotation range of motion at 3-, 6- or 12-month follow ups.

### Quality of the evidence

The rating of very low-quality evidence per outcome across trials was based on the judgement of serious limitations (risk of bias), serious imprecision and likely publication bias in all the outcomes across trials. All six trials identified in this review were rated at high risk of bias. However, we downgraded the evidence only by one level due to the fact that we did not find statistical differences between groups, suggesting that the included studies may not have been biased. Furthermore, serious indirectness was judged as an additional factor in rating down the quality of evidence for half the outcomes across trials. The very low-quality evidence synthesised limits our confidence in the effect estimates. However, given that MCID thresholds for function, pain and range of motions, as well as the 95% CI excluding these thresholds, it is unlikely that either approach to rotator cuff repair will result in superior clinical outcomes.

### Agreements / Disagreements with other reviews

The results of our systematic review and meta-analysis could not directly be compared to the findings of Shan et al. (2014), Huang et al. (2016) or Ji et al. (2015) reviews[2–4]. The Shan et al. (2014) review of 12 studies (3 RCTs, 8 retrospective studies, 1 prospective study) concluded that there were no differences in clinical outcomes of pain, function and range of motion between the arthroscopic and mini-open rotator cuff repair approaches[4]. The Huang et al. (2016) review of 18 studies (4 RCTs, 12 retrospective studies, 2 prospective study) indicated that all-arthroscopic and mini-open rotator cuff repair surgical approaches are associated with similar clinical outcomes of function, pain and range of motion and that both surgical techniques can be used interchangeably based factors such as patient and rotator tear characteristics[2]. However, it is important to note that the aforementioned reviews pooled studies to provide effect estimates irrespective of their design; RCTs were combined with prospective and retrospective observational studies. This greatly limits our confidence in the effect estimates [2,4]. Furthermore, the reviews failed to define an MCID threshold a priori, to further support their well-conducted meta-analyses and ultimately their conclusions. The Ji et al. (2015) review included 5 RCTs and concluded that there were no differences in clinical outcomes between the arthroscopic and mini-open rotator cuff repair techniques[3]. However, it is important to highlight the fact that this review pooled RCTs based on the last follow-up time point reported, which ranged from 6-months to 34-months. It is likely that the underlying reason for the high levels of heterogeneity identified in the Ji et al. (2015) review were due to the pooling of trials with such wide range of follow-ups. In addition, the review did not provide ratings of the quality of evidence and similarly failed to define an MCID threshold a priori to further support their conclusions.

Our review provides the most up-to-date state of the evidence concerning the clinical outcomes of arthroscopic vs mini-open rotator cuff repair techniques. We provided ratings of the quality of evidence according to GRADE guidelines across each outcome, included two additional large trials and provided an analysis of precision by evaluating the MCID thresholds with the 95% confidence intervals, therefore, able to make definitive conclusions for most of the included clinical outcomes. We could not provide definitive statements on whether arthroscopic approach could offer superior outcomes in terms of external rotation range of motion at 3 and 12 months because our analysis of 555 and 462 patients respectively, did not meet the criteria for our calculated Optimal Information Size of 754. As a result, it produced wider confidence intervals, therefore, MCID threshold not excluded. (S1 Fig)

Hui et al. 2017 study of 226 patients compared the immediate costs associated in patients who received mini-open and arthroscopic rotator cuff repairs and indicated that immediate costs incurred by mini-open rotator cuff technique were significantly less than those of arthroscopic technique. However, it is important to note that this was a retrospective study, and outcomes were only analysed only at 1 year follow up[32].

### Implications for research

We have limited confidence in our conclusions. Future well-designed large-scale RCTs investigating the effects of arthroscopic vs mini-open rotator cuff repair techniques on clinical outcomes of function, pain and range of motion are warranted to generate high quality evidence (i.e. greater confidence) to further ensure that the true effect lies close to that of the estimate of the effect. In addition, future cost-effectiveness trials comparing the two surgical technique are warranted.

### Implications for practice

Both arthroscopic and mini-open approaches to rotator cuff repair with post-operative rehabilitation are effective means of improving function, pain and shoulder range of motion in patients with rotator cuff tears. Despite the very-low quality synthesized, we continue to suggest that the difference between the two surgical techniques are too small to be clinically important in terms of improving clinical outcomes of function, pain and range of motion.

### Strengths & limitations

We were mainly concerned with identifying RCTs and therefore, did not included prospective or retrospective observational studies in this review. It is possible that there might be a source of publication bias within our search strategy. Two independent reviewers conducted the electronic searches in all the major databases. Furthermore, a protocol registration was undertaken prior to the conduct of this review.

## Conclusions

The effects of arthroscopic compared to mini-open rotator cuff repair, on function, pain and range of motion are too small to be clinically important at 3-, 6- and 12-month follow ups.

## Supporting information

S1 PRISMA Checklist (PDF)

S1 Table (PDF)

S1 Fig (PDF)

## Abbreviations

SRs: Systematic Reviews
RCTs: Randomized Controlled Trials
GRADE: Grading of Recommendations Assessment, Development and Evaluation
PICO: Population, Intervention, Comparator, Outcome
PRISMA: Preferred Reporting Items for Systematic Reviews and Meta-Analyses
MCID: Minimally Clinically Important Differences
SMD: Standardized Response Difference
WMD: Weighted Mean Difference
EP: Evidence Profile
SoF: Summary of Findings
SD: Standard Deviations
WORC: Western Ontario Rotator Cuff Index
DASH: Disabilities of the Arm, Shoulder and Hand
VAS: Visual Analogue Scale
