# Supplementary material for: Effects of arthroscopic vs. mini-open rotator cuff repair on function, pain & range of motion. A systematic review and meta-analysis
Source: PLoS One. 2019 Oct 31;14(10):e0222953. doi: 10.1371/journal.pone.0222953 (PMC6822715; doi:10.1371/journal.pone.0222953)
Supplement: S1 Table — (PDF) [file pone.0222953.s002.pdf]

Cochrane Risk of Bias Assessment Tool

Bias	Criteria for judging risk of bias in the Risk of Bias assessment tool
**Random Sequence Generation (Selection Bias)**	Criteria for a judgement of 'Low risk' of bias<br>The investigators describe a random component in the sequence generation process such as:<br>• Referring to a random number table;<br>• Using a computer random number generator;<br>• Coin tossing;<br>• Shuffling cards or envelopes;<br>• Throwing dice;<br>• Drawing of lots;<br>• Minimization.<br><br>Criteria for the judgement of 'High risk' of bias<br>The investigators describe a non-random component in the sequence generation process. Usually, the description would involve some systematic, non-random approach, for example:<br>• Sequence generated by odd or even date of birth;<br>• Sequence generated by some rule based on date (or day) of admission;<br>• Sequence generated by some rule based on hospital or clinic record number.<br>Other non-random approaches happen much less frequently than the systematic approaches mentioned above and tend to be obvious. They usually involve judgement or some method of non-random categorization of participants, for example:<br>• Allocation by judgement of the clinician;<br>• Allocation by preference of the participant;<br>• Allocation based on the results of a laboratory test or a series of tests;<br>• Allocation by availability of the intervention<br><br>Criteria for the judgement of 'Unclear risk' of bias<br>Insufficient information about the sequence generation process to permit judgement of 'Low risk' or 'High risk'.
**Allocation Concealment (Selection Bias)**	Criteria for a judgement of 'Low risk' of bias<br>Participants and investigators enrolling participants could not foresee assignment because one of the following, or an equivalent method, was used to conceal allocation:<br>• Central allocation (including telephone, web-based and pharmacy-controlled randomization);<br>• Sequentially numbered drug containers of identical appearance;<br>• Sequentially numbered, opaque, sealed envelopes.

	<u>Criteria for the judgement of 'High risk' of bias</u><br>Participants or investigators enrolling participants could possibly foresee assignments and thus introduce selection bias, such as allocation based on:<br>• Using an open random allocation schedule (e.g. a list of random numbers);<br>• Assignment envelopes were used without appropriate safeguards (e.g. if envelopes were unsealed or nonopaque or not sequentially numbered);<br>• Alternation or rotation;<br>• Date of birth;<br>• Case record number;<br>• Any other explicitly unconcealed procedure.<br><br><u>Criteria for the judgement of 'Unclear risk' of bias</u><br>Insufficient information to permit judgement of 'Low risk' or 'High risk'.
**Blinding of Participants And Personnel (Performance Bias)**<br>**All Outcomes**	<u>Criteria for a judgement of 'Low risk' of bias</u><br>• No blinding or incomplete blinding, but the review authors judge that the outcome is not likely to be influenced by lack of blinding;<br>• Blinding of participants and key study personnel ensured, and unlikely that the blinding could have been broken.<br><br><u>Criteria for the judgement of 'High risk' of bias</u><br>• No blinding or incomplete blinding, and the outcome is likely to be influenced by lack of blinding;<br>• Blinding of key study participants and personnel attempted, but likely that the blinding could have been broken, and the outcome is likely to be influenced by lack of blinding.<br><br><u>Criteria for the judgement of 'Unclear risk' of bias</u><br>• Insufficient information to permit judgement of 'Low risk' or 'High risk';<br>• The study did not address this outcome
**Blinding of Outcome Assessment (Detection Bias)**<br>**All Outcomes**	<u>Criteria for a judgement of 'Low risk' of bias</u><br>• No blinding of outcome assessment, but the review authors judge that the outcome measurement is not likely to be influenced by lack of blinding;<br>• Blinding of outcome assessment ensured, and unlikely that the blinding could have been broken.

	Criteria for the judgement of 'High risk' of bias<br>• No blinding of outcome assessment, and the outcome measurement is likely to be influenced by lack of blinding;<br>• Blinding of outcome assessment, but likely that the blinding could have been broken, and the outcome measurement is likely to be influenced by lack of blinding.<br><br>Criteria for the judgement of 'Unclear risk' of bias<br>• Insufficient information to permit judgement of 'Low risk' or 'High risk';<br>• The study did not address this outcome
**Incomplete Outcome Data (Attrition Bias) All Outcomes**	Criteria for a judgement of 'Low risk' of bias<br>• No missing outcome data;<br>• Reasons for missing outcome data unlikely to be related to true outcome (for survival data, censoring unlikely to be introducing bias);<br>• Missing outcome data balanced in numbers across intervention groups, with similar reasons for missing data across groups;<br>• For dichotomous outcome data, the proportion of missing outcomes compared with observed event risk not enough to have a clinically relevant impact on the intervention effect estimate;<br>• For continuous outcome data, plausible effect size (difference in means or standardized difference in means) among missing outcomes not enough to have a clinically relevant impact on observed effect size;<br>• Missing data have been imputed using appropriate methods.<br><br>Criteria for the judgement of 'High risk' of bias<br>• Reason for missing outcome data likely to be related to true outcome, with either imbalance in numbers or reasons for missing data across intervention groups;<br>• For dichotomous outcome data, the proportion of missing outcomes compared with observed event risk enough to induce clinically relevant bias in intervention effect estimate;<br>• For continuous outcome data, plausible effect size (difference in means or standardized difference in means) among missing outcomes enough to induce clinically relevant bias in observed effect size;<br>• 'As-treated' analysis done with substantial departure of the intervention received from that assigned at randomization;<br>• Potentially inappropriate application of simple imputation.<br><br>Criteria for the judgement of 'Unclear risk' of bias<br>• Insufficient information to permit judgement of 'Low risk' or 'High risk';

	• The study did not address this outcome
**Selective Reporting (Reporting Bias)**	Criteria for a judgement of 'Low risk' of bias<br>• The study protocol is available and all of the study's pre-specified (primary and secondary) outcomes that are of interest in the review have been reported in the pre-specified way;<br>• The study protocol is not available but it is clear that the published reports include all expected outcomes, including those that were pre-specified (convincing text of this nature may be uncommon).<br><br>Criteria for the judgement of 'High risk' of bias<br>• Not all of the study's pre-specified primary outcomes have been reported;<br>• One or more primary outcomes is reported using measurements, analysis methods or subsets of the data (e.g. subscales) that were not pre-specified;<br>• One or more reported primary outcomes were not pre-specified (unless clear justification for their reporting is provided, such as an unexpected adverse effect);<br>• One or more outcomes of interest in the review are reported incompletely so that they cannot be entered in a meta-analysis;<br>• The study report fails to include results for a key outcome that would be expected to have been reported for such a study<br><br>Criteria for the judgement of 'Unclear risk' of bias<br>Insufficient information to permit judgement of 'Low risk' or 'High risk'. It is likely that the majority of studies will fall into this category.
**Other Bias**	Criteria for a judgement of 'Low risk' of bias<br>Trials that did include statements on sources of funding.<br><br>Criteria for the judgement of 'High risk' of bias<br>Trials that did not include statements on sources of funding.
