# Supplementary material for: Effects of arthroscopic vs. mini-open rotator cuff repair on function, pain & range of motion. A systematic review and meta-analysis
Source: PLoS One. 2019 Oct 31;14(10):e0222953. doi: 10.1371/journal.pone.0222953 (PMC6822715; doi:10.1371/journal.pone.0222953)
Supplement: S1 Fig — (PDF) [file pone.0222953.s003.pdf]

# Optimal Information Size (OIS)

Alpha $\alpha$ error = 0.05;

Beta $\beta$ error = 0.2;

N=size per group;

$z_x$= the z-score/standard normal deviate for a two-sided x;

$\delta$ = a clinically acceptable margin;

$S^2$= Polled standard deviation of both comparison groups;

$$N = 2 \times \left( \frac{z_{1-\frac{\alpha}{2}} + Z_{1-\beta}}{\delta} \right)^2 \times s^2$$

N = 2 x (1.96 + 0.842/4.9)$^2$ x 24.0$^2$ = 377 per group.

Total = 2 x 377 = **754**
